# Bioactive Compounds, Sugars, and Sensory Attributes of Organic and Conventionally Produced Courgette (*Cucurbita pepo*)

**DOI:** 10.3390/foods10102475

**Published:** 2021-10-15

**Authors:** Klaudia Kopczyńska, Dominika Średnicka-Tober, Ewelina Hallmann, Jacek Wilczak, Grażyna Wasiak-Zys, Zdzisław Wyszyński, Katarzyna Kucińska, Aneta Perzanowska, Paweł Szacki, Marcin Barański, Paulina Gawron, Rita Góralska-Walczak, Ewa Rembiałkowska, Renata Kazimierczak

**Affiliations:** 1 Department of Functional and Organic Food, Institute of Human Nutrition Sciences, Warsaw University of Life Sciences, Nowoursynowska 159c, 02-776 Warsaw, Poland; dominika_srednicka_tober@sggw.edu.pl (D.Ś.-T.); ewelina_hallmann@sggw.edu.pl (E.H.); grazyna_wasiak_zys@sggw.edu.pl (G.W.-Z.); paulina_gawron@sggw.edu.pl (P.G.); rita_goralska_walczak@sggw.edu.pl (R.G.-W.); ewa_rembialkowska@sggw.edu.pl (E.R.); renata_kazimierczak@sggw.edu.pl (R.K.); 2 Department of Physiological Sciences, Institute of Veterinary Medicine, Warsaw University of Life Sciences, Nowoursynowska 159, 02-776 Warsaw, Poland; jacek_wilczak@sggw.edu.pl; 3 Department of Agronomy, Institute of Agriculture, Warsaw University of Life Science, Nowoursynowska 159, 02-787 Warsaw, Poland; zdzislaw_wyszynski@sggw.edu.pl (Z.W.); katarzyna_kucinska@sggw.edu.pl (K.K.); aneta_perzanowska@sggw.edu.pl (A.P.); pawel_szacki@sggw.edu.pl (P.S.); 4 Laboratory of Neurobiology, Nencki Institute of Experimental Biology, Polish Academy of Sciences, Pasteura 3, 02-093 Warsaw, Poland; m.baranski@nencki.edu.pl

**Keywords:** organic food, courgette, zucchini, *Cucurbita pepo*, sensory attributes, antioxidants, phenolics, sugars, untargeted metabolomic analysis, metabolic features

## Abstract

Organic agriculture is considered one of the elements of sustainable food production and consumption, mainly due to its limited impact on the natural environment. At the same time, the quality features of organically produced foods, especially sensory attributes and health promoting values, are important factors determining consumers’ interest, and therefore play a key role in the organic sector’s development. The aim of this study was to investigate the sensory characteristics and concentrations of sugars and selected health-promoting bioactive compounds of organic courgette compared to conventionally grown courgette. In addition, untargeted metabolomic analysis of the courgette fruits was performed. The results of this study did not show a significant effect of the horticultural system (organic vs. conventional) on the concentrations of vitamin C, carotenoids, and chlorophylls in the courgette fruits. However, the fruits from the organic systems were significantly richer in sugars when compared to the conventionally cultivated ones (*p* = 0.038). Moreover, the organic fruits fertilized with manure contained significantly higher amounts of polyphenols, including gallic acid (*p* = 0.016), chlorogenic acid (*p* = 0.012), ferulic acid (*p* = 0.019), and quercetin-3-*O*-rutinoside (*p* = 0.020) compared to the conventional fruits. The untargeted analysis detected features significantly differentiating courgette fruits depending on the cultivar and horticultural system. Some significant differences in sensory values were also identified between fruits representing the two cultivars and coming from the horticultural systems compared in the study. Conventional courgettes were characterized by the most intensive peel color and aquosity, but at the same time were the least hard and firm compared to the fruits from the two organic systems. There was also a trend towards higher overall quality of the organically grown fruits. The presented study shows that the organic and conventional courgette fruits differ in a number of quality features which can influence consumers’ health and purchasing choices.

## 1. Introduction

Agriculture is known to be one of the key sectors responsible for greenhouse gas emissions and climate change [1]. The orientation of agricultural development towards more environmentally friendly options is thus necessary [2,3,4,5]. These problems have been addressed in the 2030 Agenda for Sustainable Development of the United Nations, and the development of sustainable consumption and production patterns is a key target of SDG12.

Organic agriculture, as one of the extensive alternatives of industrial, intensive farming practices, is considered as a step towards more sustainable and environmentally friendly solutions, closely in line with the United Nations Environment Programme [5,6]. Organic practices are based on natural fertilizers, non-chemical plant protection methods, and diverse crop rotations [7,8]. Organic systems aim to minimize the impact of food production on the environment by keeping a high level of biodiversity and protecting natural resources. Although some environmental sustainability aspects of organic farming remain uncertain and are widely discussed [9], a positive impact of organic farming practices such as crop rotation on the reduction of CO_2_ emissions has been described in several reports [4]. Furthermore, organic systems clearly respond to the expectations of a growing number of consumers seeking food produced and processed in natural way. Thus, the market of organic foods is constantly growing [10]. This includes the market of fresh organic fruits and vegetables. 

At the same time, according to the current recommendations of the World Health Organization (WHO) and the Food and Agriculture Organization of the United Nations (FAO), the recommended fruits and vegetables consumption, excluding potatoes and other starchy tubers, is a minimum of 400 g (or five 80 g portions) per day [11]. Such a portion of fruit and vegetables, abundant in fiber, vitamins, and other antioxidants and low in calories, is considered an important factor for the prevention of a number of non-communicable chronic diseases, such as cardiovascular diseases, diabetes, obesity, and certain cancers [11,12,13,14]. Increasing vegetables consumption is thus seen as one of the important steps towards a healthy diet and, if associated with a reduction in the consumption of meat, is also considered as an important change towards overall diet and food system sustainability [15].

Cucurbits (*Cucurbitaceae*) is a vegetable family appreciated by farmers and consumers all over the globe [16]. One of the vegetables belonging to cucurbits is courgette (*Cucurbita pepo*) also known as zucchini or summer squash [16,17]. Courgette fruits are widely used for direct consumption as well as for processing purposes [16,18]. Along with consumers’ interest in fresh vegetables such as courgette, the demand for high-quality produce is also growing [19,20]. Quality features of organically produced foods, including sensory attributes and health promoting values, are important factors determining consumers’ interest, and therefore play a key role in the organic sector’s development. The available research on the impact of agronomic systems on the chemical composition of crops mostly shows significant differences in pesticide residue [21] and antioxidant content in organic versus conventional produce [21,22,23,24,25,26]. One of the analytical approaches increasingly used to differentiate plant raw materials and food products depending on their production and processing methods is metabolomics [27,28,29,30,31]. 

In view of the fact that the main reasons for buying organic over conventional food are their health-promoting features and higher sensory values, it seems important to conduct a chemical profile and sensory analysis of the frequently purchased vegetables, such as courgettes. The aim of this study was therefore to perform the chemical analysis and quantitative descriptive analysis (QDA) of the sensory features of organic and conventional courgette fruits of two cultivars (Nimba and Astra Polka). A targeted analysis concentrated on sugars and the determination of bioactive compounds (polyphenols, carotenoids, chlorophylls, and vitamin C). In addition, an untargeted metabolomic analysis was performed. This metabolomic analysis, without identifying specific low molecular weight compounds, was used as a supplementary tool for the targeted analysis of the qualitative features (sensory and chemical) in order to verify its potential for differentiating samples depending on their genotype and production system.

## 2. Materials and Methods

### 2.1. Study Design and Plant Material

In this study, fruits of two popular cultivars (Nimba and Astra Polka, Figure S1 in the Supplementary Materials) of courgette were harvested from three horticultural systems: two organic, based on (a) manure or (b) commercial organic pelleted multi-component fertilizer, and one conventional, with standard mineral fertilization. Courgettes were grown in 2018 in a controlled field trial in the Experimental Field of the Department of Agronomy, Warsaw University of Life Sciences, Miedniewice, Poland (51°57′ N 20° 11′ E). The plots of the organic horticultural system were certified by “AGRO BIO TEST”. Technologically mature courgette fruits were harvested three times in the season: at the beginning of July, at the turn of July and August, and at the end of August. 

The Nimba and Astra Polka cultivars have very similar morphological features. Their fruits are characterized by an elongated and cylindrical shape which is narrow at the stem. Their skin is thin and dark green, with a fair pattern characteristic of Nimba, and light green tiny spots typical of Astra Polka.

### 2.2. Preparation of Samples

Fruits collected randomly from different locations within the field (5 kg from every horticultural system and cultivar combination) were delivered to the Department of Functional and Organic Food of the Warsaw University of Life Sciences. Fresh fruits were washed and cut into cubes (5 cubic mm) from which about 20 g was used for the analysis of dry matter content. Another 100 g of fresh fruit samples were deep frozen (24 h, −80 °C) and then freeze-dried at −40 °C and 0.100 mBa of pressure (Labcono 2.5 freeze-dryer, Labcono Corporation, Kansas City, MO, USA). Freeze-dried fruit samples were ground in the laboratory mill (IKA^®^-Werke GmbH & Co. KG, Staufen im Breisgau, Germany), placed in scintillation vials, and stored (−80 °C) for further analyses. This method of the preparation of samples was previously described by Średnicka-Tober et al. [32]

For the sensory analyses (quantitative descriptive analysis, QDA), around 3 kg of fruits from each combination of cultivar and horticultural system were washed and cut into slices of 1 cm of thickness and no more than 5 cm of diameter. Courgette slices were placed on a sieve and steamed over the boiling water for 5 min, and then cooled at room temperature. Every QDA expert received two courgette slices from each horticultural system and cultivar.

### 2.3. Chemicals

HPLC grade acetone, ethyl acetate, and meta-phosphoric acid were obtained from Sigma-Aldrich (Poznań, Poland). HPLC grade acetonitrile, HPLC grade methanol, ortho-phosphoric acid, and ultrapure magnesium carbonate were obtained from Chempur (Piekary Śląskie, Poland). Standards of *β*-carotene, caffeic acid, chlorogenic acid, chlorophyll *a*, chlorophyll *b*, dehydroascorbic acid, ferulic acid, gallic acid, kaempferol-3-*O*-glucoside, lutein, *L*-ascorbic acid, *p*-coumaric acid, and quercetin-3-*O*-rutinoside (HPLC grade, 99.5–99.9% pure) were obtained from Fluka and Sigma-Aldrich (Poznań, Poland). Standards of fructose (99.9% CAS 57-48-7), glucose (99.9% CAS 50-99-7), and sucrose (99.9% CAS 57-50-1) were obtained from Sigma-Aldrich (Warsaw, Poland). 

### 2.4. Dry Matter

The dry matter content analysis was carried out according to the Polish Norm PN-EN-12145:2001 [33]. Courgette samples in cubes were weighed and hot air-dried in 105 °C for 72 h (FP-25W Farma Play dryer, from Farma Play, Marki, Poland), and then cooled in desiccators at room temperature and weighed again. The content of dry matter was calculated by difference of mass before and after drying and expressed in grams in 100 g of fresh matter (f.w.).

### 2.5. Targeted Analysis of Polyphenols-Extraction and Determination

The content of phenolic compounds in courgette fruit samples was determined by the high performance liquid chromatography (HPLC) method, as previously described by Średnicka-Tober et al. [32]. The 100 mg samples of courgette powder were weighed and combined with 5 mL of 80% methanol (*v*/*v* aqueous solution), shaken by a Micro-Shaker 326 M (Premeo, Marki, Poland), and ultrasonicated for 10 min at 30 °C and 5500 Hz [34]. The next step was the centrifugation of the samples for 10 min at 6000 rpm and 0 °C. Then, 1 mL of supernatant was transported into vials and analyzed by HPLC. The same HPLC system (Shimadzu, USA Manufacturing, Inc., Canby, OR, USA: two pumps LC-20AD, controller CBM-20A, column oven SIL-20AC, spectrometer UV/Vis SPD-20 AV) was used in this and all other targeted analyses described in the following sections. The volume of injection was 100 µL. The separation of polyphenols was conducted with the Synergi Fusion-RP 80i Phenomenex column (250 × 4.60 mm) under gradient conditions with a flow rate of 1 mL min^−1^. The gradients of phase A and phase B were as follows: 10% (*v*/*v*) acetonitrile and ultra-pure water (phase A) and 55% (*v*/*v*) acetonitrile and ultrapure water (phase B). The ortho-phosphoric acid (pH 3.0) was used for acidification. The wavelength used for detection was 270 nm (for phenolic acids detection) and 360 nm (for flavonoids detection). The external standards of polyphenols with purities of 95.00–99.99% were used for the identification of compounds. The concentrations of polyphenols were calculated based on standard curve and sample dilution coefficients.

### 2.6. Targeted Analysis of Carotenoids and Chlorophylls: Extraction and Determination

Carotenoids and chlorophylls extraction and identification was carried out as previously described by Nishiyama et al. [35], with small modifications. The freeze-dried courgette samples (100 mg) were interfused with pure acetone (5 mL) and mixed by a vortex agitator. Then, samples were ultrasonicated for 15 min at the temperature of 0 °C and centrifuged (6000 rpm, 10 min, 0 °C). Next, the supernatant was collected in dark glass vials. The Max-RP 80A column (250 × 4.6 mm) was used for the separation of compounds. The injection volume was 100 µL and detection was performed under 445 and 450 nm wavelengths for 18 min. The quantification of the compounds was based on external standards.

### 2.7. Targeted Analysis of Vitamin C: Extraction and Determination

The method of vitamin C (*L*-ascorbic acid (L-ASC) and dehydroascorbic acid (DHA)) extraction and identification was previously described in Kopczyńska et al. [36]. The dried material (100 mg) was mixed by vortex with 5 mL of 5% meta-phosphoric acid. After ultrasonication (15 min, 20 °C) and centrifugation (6000 rpm, 10 min, 0 °C), samples were analyzed in the HPLC device. The separation of compounds was performed with the use of a Phenomenex Hydro 80-A RP (250 × 4.6 mm) column and a mobile phase of 50 mM phosphate buffer (pH 4.4.) and 0.1 mM sodium acetate. Wavelengths of 255 and 260 nm were used for detection. The standards of L-ASC and DHA were used for the quantification of the compounds.

### 2.8. Targeted Analysis of Sugars: Extraction and Determination

The sugars extraction and identification method used in this study was previously described by Ponder and Hallmann [37]. The freeze-dried materials (100 mg) of courgette fruits and acetone (80%, 5 mL) were mixed. Ultrasonication (10 min, 0 °C) and centrifugation (6000 rpm, 10 min, 0 °C) were used. The supernatant was collected and transferred to the vials. The fructose, glucose, and sucrose contents were analyzed using the HPLC system with a RID-10A detector. The fructose, glucose, and sucrose were identified and separated using a Phenomenex Luna NH2 Chromatography Column (Phenomenex, Shimpol, Warsaw, Poland) under isocratic conditions with a flow rate of 1 mL min^−1^, using 80% acetone with deionized water. The analysis time was 15 min. The respective sugars were identified by the analysis of the retention times of external standards. 

### 2.9. Quantitative Descriptive Analysis (QDA)

The quantitative descriptive analysis (QDA) of the courgette fruits was conducted in the accredited Laboratory of Sensory Analysis in the Chair of Functional Food and Sensory Research of the Warsaw University of Life Sciences (Poland) according to ISO standard 13299:2016 [38]. The qualitative sensory attributes for the evaluation were selected by a group of experts and are presented in Table 1. The unstructured linear scale with marked marginal values from 0 to 10, where 0 means no intensity of a given attribute, and 10 means high intensity of a given attribute, was used in this study. The ANALSENS computer software was used for the evaluations. The analyses were performed in the laboratory with separated evaluation sites, standard lights, and controlled temperature and relative humidity. The evaluation of each sample was carried out by eight qualified experts, in two sessions, according to PN-EN ISO 8586-03:2014 [39]. The unit samples (two courgette slices each) were placed in previously prepared and coded plastic containers (150 mL) with lid covers. The unsweetened tea (temperature around 50 °C) was served to neutralize the taste between each of the evaluated samples.

### 2.10. Untargeted Analysis of Metabolic Features: Extraction and Detection

The extraction of the samples for untargeted metabolomic analysis was initiated by mixing 100 mg of freeze-dried courgette powder by vortex with 2.5 mL of methanol (99% purity) and 2.5 mL of acetonitrile (99% purity). Next, samples were centrifuged (13,000 rpm, 15 min, 0 °C) and the supernatant was transferred into glass vials. The detection was performed using the Symbiosis Pico UHPLC system. Chromatographic separation was performed by the reversed-phase method on a Hypersil chromatographic column, BDS C18, 150 × 4.6 mm, 5 mm with a Hypersil C18 guard column (10 × 2.1 mm, size 5 μm). The mobile phase consisted of (A) methanol:formic acid (99:1, *v/v*) and (B) water:formic acid (99:1, *v*/*v*) and the flow rate was constant at 500 µL min^−1^. The gradient elution of the mobile phase started with 100% A, then proceeded in the following order: 1.1–40 min, linear gradient to 100% B; 40.1–55 min, linear gradient to 100% B; and 55.1–60 min, linear gradient to 100% A. The runtime of the method was 60 min. A SCIEX TripleTOF 5600+ DuoSpray Source for a SCIEX TripleTOF 5600+ (TurboIonSpray) detector was used. MS parameters were as follows: the optimized detection conditions included curtain gas (N_2_) at 25 psi, nebulizer gas (N2) at 20 psi, heater gas (N_2_) at 50 psi, ion source voltage floating at 5500 V, and source temperature at 500 °C. Samples were measured with a heated electrospray ionization probe in positive ionization (H-ESI+). Every third sample analyzed using the Calibrant Delivery System (SCIEX) MS system was auto-calibrated using original calibrators (SCIEX). Metabolic feature profiles obtained in the 100–1100 Da range with 5 cps sensitivity were analyzed using SCIEX MarkerView^TM^ and XCMSplus software. The generated metabolomics profiling data sets were processed by the control software of the Analyst^®^ mass spectrometer and saved in a specific data format (*.raw). The first step was to convert data from Excalibur-specific raw files to open format files (*.mzXML) using MS Convertor software (MSConvert). Subsequently, metabolomics data were processed using the XCMSplus platform.

### 2.11. Statistical Analyses

The statistical analyses of the chemical composition and sensory attributes data were carried out in the R statistical environment [40]. The two-factor analyses of variance (ANOVA) were performed using a linear-mixed effects model, with the courgette cultivar and horticultural system as fixed effects, and field replication or assessor number as a random effect factor. This allowed the significance of the effects of the experimental factors and their interactions to be tested. The significance of the differences between the interaction means was tested using Tukey’s HSD post hoc test. Before the parametric analyses, the normality of the data distribution was verified using the qqnorm function in R. No data transformation was required. The principal component analysis (PCA) and redundancy analysis (RDA) were carried out using the “vegan” package library in R, to further explore possible differences and similarities in the chemical composition and sensory characteristics of the courgette fruits of two cultivars, grown under different horticultural management systems. Additionally, Pearson’s product-moment correlation analyses were performed to identify potential linear associations between the individual sensory attributes using the ‘cor’ function in R and visualized with the use of the ‘corrplot’ package.

The results of the untargeted metabolomic analyses of the courgette fruits of two cultivars, grown in different horticultural systems, underwent principal component analysis (PCA) and were compared using Student’s t-tests and fold change rate in MarkerView™ software.

## 3. Results and Discussion

### 3.1. Dry Matter and Sugars in Courgette Fruits

The tested courgette fruits contained, on average, 4.29 g of dry matter in 100 g of fresh weight. The two-factor ANOVA showed no significant impact of the growing system and cultivar on the dry matter content in the fruits (Table 2). Other published research shows similar ranges of dry matter content in fruits of *Cucurbita pepo* [41]. However, different cultivars and morphological parts (mesocarp and epicarp) were shown to significantly differ in the dry matter content [41]. A higher amount of dry matter was found in fruits belonging to *Cucurbita moschata* and *Cucurbita maxima* [42]. Other authors confirm the high importance of plant genotype as a factor differentiating dry matter content in courgettes [43].

The content of fructose, glucose, sucrose, and the sum of these three sugars was also analyzed in the courgette fruits. The total content of all three sugars was higher in the fruits cultivated in the organic system with commercial organic fertilizer and manure than in the fruits cultivated conventionally. At the same time, the two tested courgette cultivars did not differ significantly in the sugars content (Table 2). Fructose was a major sugar in the tested fruits, with an average content of 1.85 g per 100 g f.w., whereas the average glucose and sucrose contents were 0.95 g and 1.00 g in 100 g f.w., respectively. When comparing the three production systems, sucrose content was highest in the fruits fertilized with manure, while glucose was highest in the fruits grown in the system with commercial organic fertilizer (Table 2). No significant interactions between the effect of the cultivar and horticultural system on the sugars content in the tested fruits were observed. Compared to the presented results, higher average sugars content was reported by other authors in the fruits of *Cucurbita maxima* [42], *Cucurbita pepo* [44,45], and *Cucurbita moschata* D. [46]. Other studies reported that courgette fruits also contain galactose, raffinose, stachyose, and starch. Fructose content was quantitatively dominant among all sugars in the courgette fruits, independently of the cultivar. In contrast to the presented results, cultivar was shown in other studies to have a significant effect on carbohydrates content in *Cucurbita pepo* [44] and *Cucurbita moschata* D. species [46]. At the same time, the studies on the carbohydrates content in plants depending on the cultivation system do not show consistent trends. Some authors have reported no significant production system effects on the content of these compounds [46], while others confirm that carbohydrates content in *Cucurbita pepo* was increased with the use of higher NPK conventional fertilizer doses [47]. The results of the largest published meta-analysis, comparing the composition of organic vs. conventionally produced crops and foods, confirmed, on average across various crop species, seasons, and production regions, a significantly higher content of carbohydrates and reducing sugars in organic compared to conventional crops [21].

### 3.2. Vitamin C and Phenolics in Courgette Fruits

The courgette fruits within this study were also tested for dehydroascorbic acid (DHA) and L-ascorbic acid (L-ASC) contents. Neither the cultivar nor the production system has shown to be a significant factor affecting the contents of these two compounds (Table 3). The average content of dehydroascorbic acid in the fruit was higher than the content of L-ascorbic acid (6.34 mg vs. 1.38 mg in 100 g f.w., respectively). Courgette fruits are not considered a rich source of vitamin C, and therefore the analysis of vitamin C content in courgettes is generally not common. Other authors reported higher ascorbic acid content in organic compared to conventional *Cucurbita maxima* D. [48] and no differences comparing organic and conventional *Cucurbita moschata* D. [46]. It was previously reported that organic crops tend to be generally richer in vitamin C compared to conventional crops [21], which is especially seen in the case of those commonly known to be rich in vitamin C.

Phenolic compounds content in the courgette fruits was also analyzed in this study. Overall, five phenolic acids were identified in the courgette fruits. Gallic acid was dominant (with an average content of 13.83 µg g^−1^ f.w. in all tested courgette samples), followed by *p*-coumaric, chlorogenic, ferulic, and caffeic acids. The identified flavonoids included quercetin-3-*O*-rutinoside and kaempferol-3-*O*-glucoside (Table 4). 

The organic courgette fruits contained higher amounts of polyphenols, including phenolic acids (sum) and flavonoids (sum), compared to the conventional fruits, but only when fertilized with manure. The organic fruits grown with commercial organic fertilizer were characterized by lower contents of phenolics, comparable with the conventional, minerally fertilized fruits (Table 3). A similar trend was true for most of the individual phenolic acids and flavonoids identified in the courgette fruits within the study (Table 4). At the same time, the ANOVA showed no significant cultivar effect on the content of the tested phenolic compounds (Table 3 and Table 4).

The impact of the growing system on cucurbits cultivation and quality features such as phenolics concentration were previously analyzed by some authors. Organic courgette fruits were shown to contain higher amounts of phenolic acids [49] and flavonoids [36,50] compared to conventional fruits, which is generally in line with the currently obtained data.

Nitrogen availability to plants as well as irradiation were reported to be strong agronomic and environmental drivers for polyphenols concentration in recent studies comparing the effect of organic and mineral fertilizers on phenolic levels in wheat [51,52]. However, in the study reported here, the nitrogen availability pattern was not monitored, and it was therefore impossible to investigate the relation between this potential driver and phenolic levels. Previous studies with grapes, wheat, and potatoes also indicated that variety choice is a major explanatory variable for phenolics concentrations [22,53,54]. Diseases and pests pressure during plant cultivation can also result in an increase of phenolics concentrations, since these plant secondary metabolites are known to be involved in the plants’ inducible response against such biotrophic stress factors [55]. Higher pest and disease incidence has been previously described as one of the main reasons for higher levels of resistance to phytochemicals contents in organic compared to conventional plant crops [56], even though there is no experimental evidence [51,52].

Concentrations of polyphenols in fruits of *Cucurbita pepo* and *Cucurbita moschata* are generally low compared to those found in *Cucurbita maxima* [17,57]. Bitter melon, which belongs to cucurbits, better known in tropical regions, is also more abundant in polyphenols compared to courgettes [58]. The variability in the contents of polyphenols in cucurbits of different genotypes, including courgettes, was previously reported [17,49,59].

### 3.3. Carotenoids and Chlorophylls in Courgette Fruits

The content of *β*-carotene, lutein, and zeaxanthin in courgette fruits was analyzed within this study. Neither the cultivation system nor genotype (cultivar) showed a significant effect on the concentration of these carotenoids in the courgette fruits (Table 5). *β*-carotene was found to be a dominant carotenoid in the tested fruits. Different species within the *Cucurbitaceae* family are characterized by various profiles and contents of carotenoid compounds, many of them much higher than those found in the tested courgettes [17,46,57]. High carotenoids concentrations are especially typical for orange pulp cucurbits [59]. In contrast to the presented results, many studies also reported significant differences in the carotenoids content depending on the cultivar of particular species [17,41,60]. When comparing crops from different agronomic systems, higher quantities of carotenoids were found, on average, in organic compared to conventional crops [21,61], including pumpkin [48].

The chlorophyll (chlorophyll *a* and chlorophyll *b*) contents in courgette fruits were also analyzed and, similar to carotenoids, none of the factors had a significant impact on chlorophyll *a* and chlorophyll *b* content (Table 5). The chlorophyll *a* content was higher than that of chlorophyll *b* (1.62 mg vs. 0.51 mg in 100 g f.w. on average in all courgette fruits, respectively). Even though chlorophylls belong to valuable antioxidants [62], their analysis in vegetables, such as courgettes, is rare. Some authors reported a significant impact of courgette cultivar on chlorophylls concentration [63]. Although it is known that the organic way of growing increases the production of many antioxidants in plants, the chlorophyll content of organic vegetables is not often discussed. Higher chlorophyll content is usually discussed as the reaction of plants to oxidative stress [64].

### 3.4. Principal Component Analysis: Chemical Composition of Courgette Fruits

**Figure 1:**
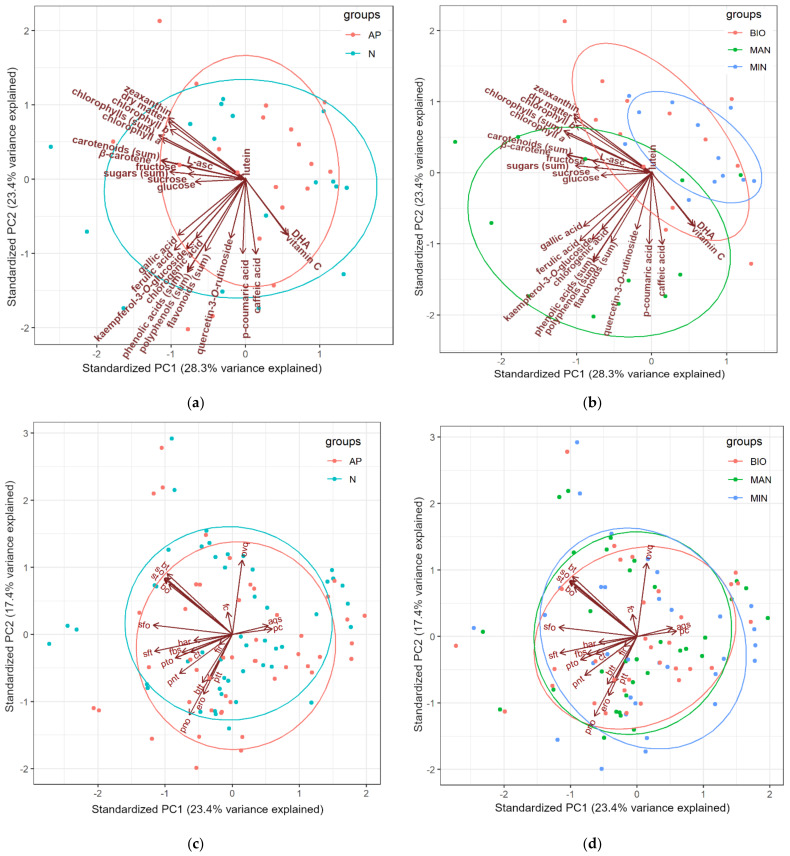
Principal component analysis (PCA) biplots of data on chemical composition, sugars and bioactive compounds of fruits of Astra Polka (AP) and Nimba (N) cultivars, (**a**) grown in horticultural system with commercial organic fertilizer (BIO), manure (MAN), and mineral (MIN) fertilizer, (**b**) as well as data on sensory attributes of fruits of (**c**) both cultivars and (**d**) grown with three fertilizers. PC1, the first principal component; PC2, the second principal component; Bo, buttery odor; pto, potato odor; so, sweet odor; sfo, sunflower odor; pno, pungent odor; ero, earthy odor; ptt, potato taste; sft, sunflower taste; bt, buttery taste; st, sweet taste; ct, cucumber taste; btt, bitter taste; pnt, pungent taste; pc, peel color; fc, flesh color; har, hardness; fir, firmness; aqs, aquosity; fbs, fibrousness; ovq, overall quality.

A principal component analysis (PCA) was performed to further explore possible differences and similarities in the composition of the courgette fruits representing different cultivars (Figure 1a) and grown in different horticultural systems (Figure 1b). While the PCA ellipses based on the data for the two courgette cultivars clearly overlap each other, those of the three horticultural systems show a clearer separation. The PCA plot also shows a positive association between the horticultural system based on organic fertilization (manure) and concentrations of a majority of the tested compounds, mainly phenolics (Figure 1b). A similar association was not observed between the cultivar and fruit composition parameters when the results for the two cultivars were plotted (Figure 1a).

### 3.5. Sensory Features of Courgette Fruits

**Figure 2:**
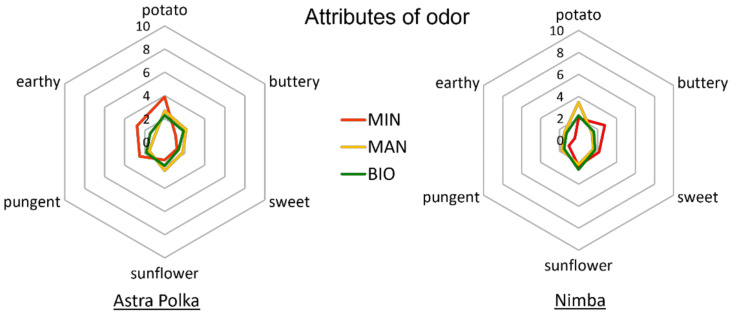
Intensity of odor attributes of courgette fruits of Astra Polka and Nimba cultivars grown in horticultural system with commercial organic fertilizer (BIO), manure (MAN), and mineral (MIN) fertilizer.

**Figure 3:**
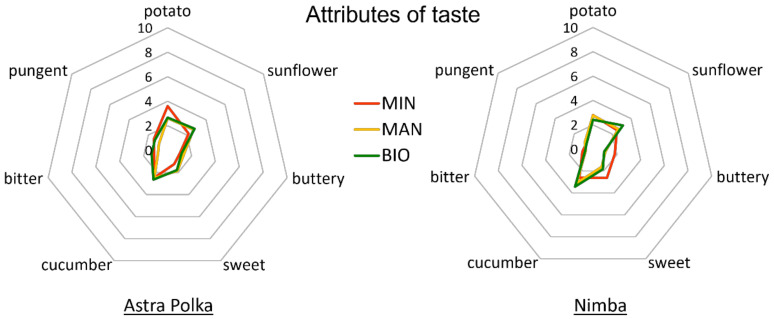
Intensity of taste attributes of courgette fruits of Astra Polka and Nimba cultivars grown in horticultural system with commercial organic fertilizer (BIO), manure (MAN), and mineral (MIN) fertilizer.

**Figure 4:**
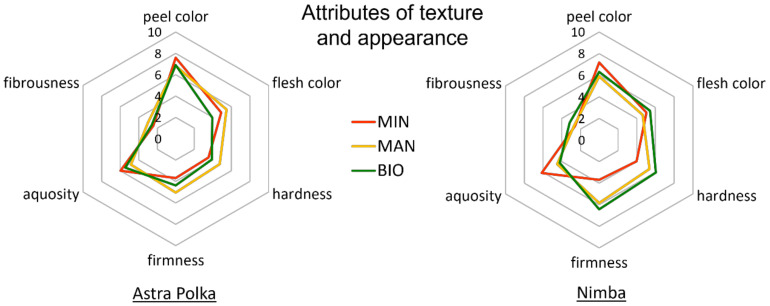
Intensity of texture and appearance attributes of courgette fruits of Astra Polka and Nimba cultivars grown in horticultural system with commercial organic fertilizer (BIO), manure (MAN), and mineral (MIN) fertilizer.

Sensory features, including taste attributes, are crucial for consumers’ view on foods. The evaluation of sensory attributes gives valuable information for the development of new food products and the improvement of existing ones [65]. The results of the sensory characteristics of the courgette fruits tested within the present study, including odor, taste, appearance, texture, and overall quality, are presented in Table 6, Table 7 and Table 8 and Figure 2, Figure 3 and Figure 4 and Figures S2–S4 (in the Supplementary Materials). The horticultural system appeared to have a significant effect on the potato odor of the courgette fruits (Table 6).

Fruits from the organic system based on manure were characterized by the most intensive potato odor, in contrast to the fruits fertilized by the commercial organic fertilizer. The fruits of the two tested cultivars differed significantly in pungent and earthy odor. These odors were perceived as stronger in fruits of the Astra Polka cultivar compared to those of the Nimba cultivar. However, significant interactions between the cultivar and horticultural system were observed in the case of buttery, potato, pungent, and earthy odor (Table 6). In the case of Astra Polka, conventional production resulted in the most intensive potato, pungent, and earthy odor, but the least intensive buttery odor, compared to the two organic systems, while in the case of Nimba, the production system effect was the opposite (Figure S2 in Supplementary Materials). The ANOVA showed no significant impact of any of the two factors on the intensity of sweet and sunflower odor of the courgette fruits (Table 6).

The ANOVA showed no significant impact of the horticultural system on the perceived taste attributes of the courgette fruits. At the same time, fruits of the two studied cultivars differed significantly in the intensity of sweet, cucumber, and pungent taste (Table 7). 

Nimba fruits were characterized by significantly more intensive sweet and cucumber taste, but less intensive pungent taste compared to the Astra Polka fruits. However, significant interaction between the cultivar and horticultural system was observed in the case of sweet taste (Table 7). In the case of Nimba, conventional production resulted in the most intensive sweet taste compared to the two organic systems, while in the case of Astra Polka, the intensity of sweet taste was lowest in the fruits coming from the conventional system (Figure 3 and Figure S3 in the Supplementary Materials).

The analysis of variance showed the significant impact of the horticultural production system on attributes such as peel color, hardness, firmness, and aquosity in the courgettes. The conventional courgettes were characterized by the most intensive peel color and aquosity, but at the same time were the least hard and firm compared to the fruits from the two organic systems (Table 8). There was also a trend towards lower overall quality of the conventionally grown fruits (significant only in the case of the Astra Polka cultivar) (Figure S4 in Supplementary Materials). Fruits of the two cultivars differed significantly in their hardness and firmness, with those of the Nimba cultivar being generally characterized by higher values of these two attributes. Other attributes, such as flesh color and fibrousness, did not significantly differ between fruits representing the different cultivars and/or production systems (Table 8).

A principal component analysis (PCA) was also performed to further explore possible differences and similarities in the sensory characteristics of courgette fruits representing different cultivars (Figure 1c) and grown in different horticultural systems (Figure 1d). The PCA ellipses of data on the sensory attributes of the different courgette cultivars and different production systems overlap each other, and thus do not show any clear differentiation of the fruit sensory profiles based on the studied experimental factors.

According to the previously published reports, the differences in sensory attributes between organic and conventional crops and foods are not always noticeable [66]. The sensory quality of fruits, vegetables, and grains may depend on their cultivar, weather conditions during cultivation, farming practices, and a number of other factors [67]. Talavera-Bianchi et al. (2010) [68], in their study on the relation between the development stage, sensory properties, and volatile content of organically and conventionally grown pac choi, have found that the differences in sensory characteristics among stages of growth were generally more substantial than the differences related to the organic and conventional production. Their results also suggest that the effect of organic production on sensory characteristics may be more evident at early stages of plant development. Rodriguez et al. (2001) [69], in their study on the sensory evaluation of fresh tomatoes from conventional, integrated, and organic production, did not detect any significant differences among the three farming system treatments for any of the tested attributes of flavor and taste. Heeb et al. (2005) [70] reported a significant impact of nitrogen form on the taste of tomatoes, with higher scores of sweetness, acidity, flavor, and overall acceptance of tomatoes grown with the organic or the ammonium-dominated treatments compared with those grown with the nitrate dominated nutrient solution. Raffo et al. (2014) [71] highlighted a significant effect of fertilization treatments (organic vs. conventional) on 10 of 14 measured sensory attributes of Golden Delicious apples. In particular, fertilization treatments significantly affected green, citrus, and floral odors; sweet and sour taste; and overall fruity and green flavors; hardness; and mouthfeel, even though a season to season variability was also marked. Casals et al. (2018) [72] pointed to the significant impact of grafting on the sensory profiles of tomatoes grown in conventional and organic management systems. Paolo et al. (2019) [73] reported that the effect of organic growing on the sensory profiles of dried tomatoes depends strongly on the drying techniques. The authors underlined that organic products are often processed in order to prolong their shelf-life; thus, research efforts on comparing organic and conventional processed foods, and not only raw materials, are of growing relevance in the food science.

**Figure 5:**
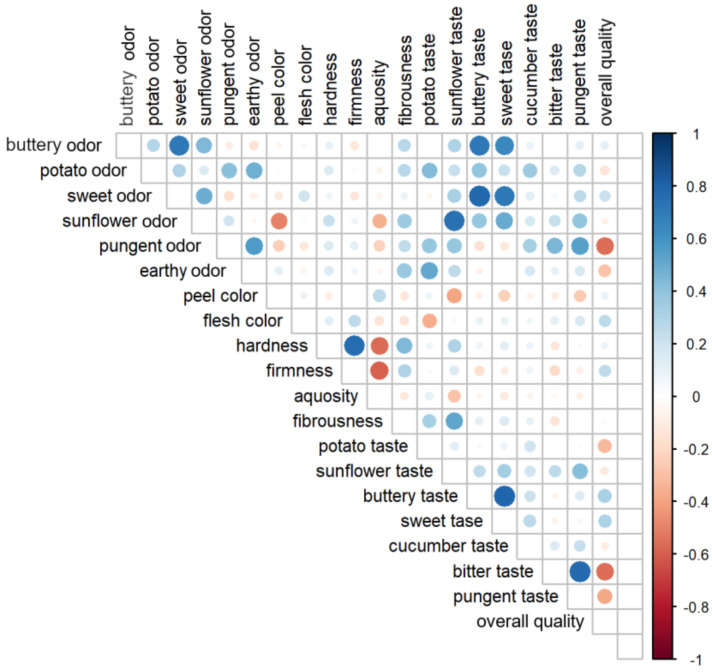
Pearson’s correlations between sensory attributes of courgette fruits. Color (red/blue) and the color intensity indicate the direction (positive/negative) and the strength of the correlation, while the size of the circle reflects the statistical significance of the correlation (*p*-value).

The Pearson’s correlation analysis of individual taste, odor, appearance, and texture features was also performed in the present study and its results are shown in Figure 5. The pungent odor, pungent taste, and bitter taste were found to be associated with the lower overall quality of fruits. The positive correlations between buttery and sweet odor, buttery and sweet taste, bitter and pungent taste, sunflower taste and odor, and hardness and firmness were also noted (Figure 5). At the same time, a negative association was identified between sunflower odor and peel color of the fruit. According to the literature, sweet taste was found to positively correlate with the overall sensory quality assessment of plants belonging to *Cucurbitaceae* [42]. In pumpkins, bitter taste was found to be negatively correlated with overall quality assessment, which is in line with the presented results. Furthermore, other authors reported significant differences in the sensory attributes of plants representing different genotypes (cultivars) [74], which was also shown in the present study in the case of a number of tested sensory attributes.

### 3.6. The Relationship between the Sensory Features and Chemical Composition of Courgette Fruits

**Figure 6:**
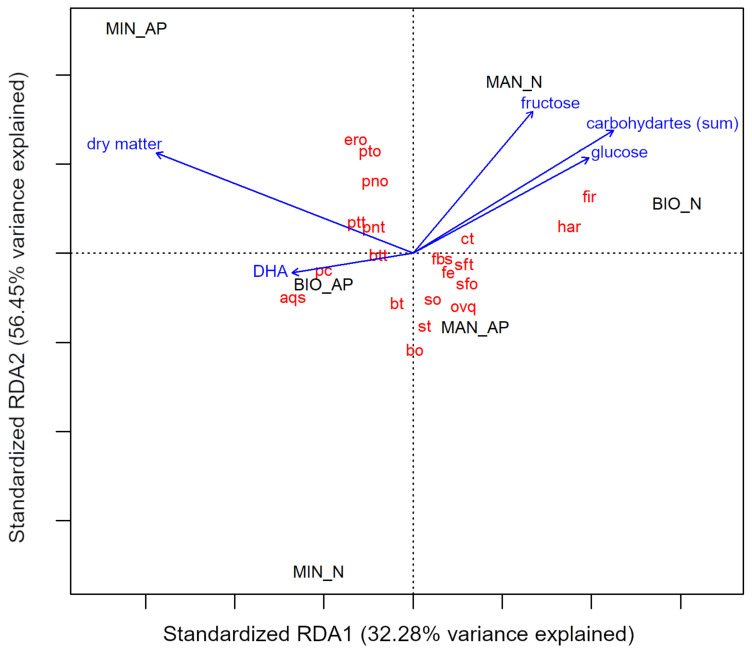
The redundancy analysis (RDA) biplot of chemical composition parameters (sugars, bioactive compounds) and sensory attributes of courgette fruits of Astra Polka (AP) and Nimba (N) cultivars grown in horticultural system with commercial organic fertilizer (BIO), manure (MAN), and mineral (MIN) fertilizer. Bo, buttery odor; pto, potato odor; so, sweet odor; sfo, sunflower odor; pno, pungent odor; ero, earthy odor; ptt, potato taste; sft, sunflower taste; bt, buttery taste; st, sweet taste; ct, cucumber taste; btt, bitter taste; pnt, pungent taste; pc, peel color; fc, flesh color; har, hardness; fir, firmness; aqs, aquosity; fbs, fibrousness; ovq, overall quality.

The redundancy analysis (RDA) was performed to study the relationships between the chemical composition parameters and sensory attributes of courgette fruits (Figure 6). The content of DHA, dry matter, fructose, glucose, and sum of sugars were identified as the main drivers for the tested sensory attributes of the courgette fruits. A positive association was observed between dry matter content and earthy odor, potato odor, pungent odor, potato taste, and pungent taste. This relation was characteristic for conventional fruits of the Astra Polka cultivar. The contents of sugars (sum), fructose, and glucose were found to be positively associated with hardness and firmness, especially for the organic Nimba fruits. The positive relation between DHA content and sensory attributes such as intensity of peel color and aquosity was also observed. The RDA analysis also shows that the organic samples of the Nimba and Astra Polka cultivars are located in different quarters of the biplot, which indicates substantial differences between the organic fruits depending on the cultivar.

The obtained results confirm that the sensory features of the courgette fruits depend on dry matter and sugars content. A strong correlation of sweet taste, determining overall quality assessment, with sugars, dry matter, and carotenoids contents was previously shown in fruits of other varieties of cucurbits [42,74]. The relation between individual taste attributes and overall quality assessment depending on the cultivars of cucurbits was also previously shown [42]. A correlation between chlorophyll content and the color and firmness of courgette fruits cut in slices was also reported [63].

### 3.7. Untargeted Metabolomic Analysis of Organic vs. Conventional Courgette

**Figure 7:**
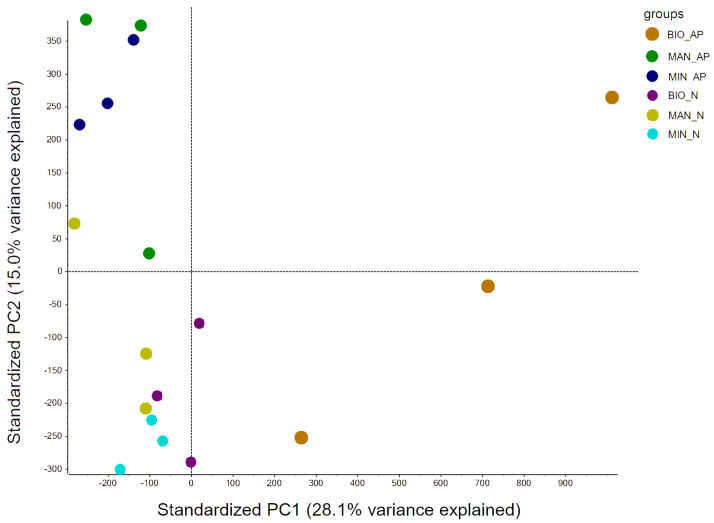
A principal component analysis (PCA) biplot of data on metabolic features of courgette fruits of Astra Polka (AP) and Nimba (N) cultivars grown in horticultural systems with commercial organic fertilizer (BIO), manure (MAN), and mineral (MIN) fertilizer. PC1, the first principal component; PC2, the second principal component.

The untargeted metabolomic analysis allowed for the detection of around 1000 different metabolic features in the tested courgette fruits. The principal component analysis (PCA) was performed to explore possible differences and/or patterns in the metabolomic profiles of courgette fruits representing different cultivars and grown in different horticultural systems (Figure 7). While data for Astra Polka fruits grown with commercial organic fertilizer were the most scattered, the results representing the other combinations of horticultural systems and cultivars were clearly grouped in the PCA biplot.

The undertaken untargeted analysis allowed for the detection of several metabolic features significantly differentiating the courgette fruits of different cultivars and coming from different horticultural systems. Nimba fruits were characterized by 154 metabolic features significantly differentiating them from Astra Polka fruits. The number of metabolic features significantly differentiating courgette fruits depending on the horticultural system were 119, 125, and 127 for the following comparisons: mineral fertilizer vs. commercial organic fertilizer, mineral fertilizer vs. manure, and manure vs. commercial organic fertilizer, respectively. Information (i.e., retention time) about the metabolic features for which the m/z values (the ratio of the atomic mass to the number of elementary ion charges) varied most significantly between the compared fruits are presented in Table S1 in the Supplementary Materials. The difference in the m/z values of these metabolic features in the compared courgette fruits is presented as a fold change. The highest fold change was noted for a group of metabolic features differentiating fruits cultivated in the conventional system from fruits grown in the organic system with the use of commercial organic fertilizer (fold change = 243.30, *p* = 0.0000).

Metabolomics was previously tested in some studies as a potentially useful tool for the authentication of crops grown in organic production systems [75,76,77,78], as well as for recognition of the geographical origins or cultivars of various crops [79,80] and their processing methods [81,82]. Omics technologies, including metabolomics, have allowed for the detection of possible nutritional differences between organic and conventional production, although many results remain uncertain [83].

The differences in quantity and type of metabolites are reflected in many food features, including sensory attributes. For example, sensory profiles of melon fruits (*Cucumis melo.*, belonging to the *Cucurbitaceae* family) depend on metabolites of esters, alcohols, aldehydes, ketones, lactones, terpenoids, and sulfur compounds [84,85]. The bitter and spicy taste of dill was reported to be related to the accumulation of several organic acid and amino acid metabolites [86].

## 4. Conclusions

Organic farming aims to provide food produced in accordance with the sustainable use of resources and with the high-quality properties valued by consumers. The presented study focused on qualities such as the health-promoting bioactive compounds, sugars, and sensory attributes of courgette, the vegetable consumed worldwide and performing well in organic horticultural systems. The obtained results did not show a significant effect of the horticultural production system (organic vs. conventional) on the concentration of vitamin C, carotenoids, and chlorophylls in the courgette fruits. However, fruits from both organic systems were generally richer in sugars compared to the conventional fruits. At the same time, organic courgette fruits contained significantly higher amounts of health-promoting plant secondary metabolites–polyphenols, including phenolic acids and flavonoids–compared to the conventional fruits, but only when fertilized with manure. Organic fruits grown with commercial organic multi-component fertilizer were characterized by a lower content of phenolics comparable to that of conventional, minerally fertilized fruits. A similar trend was true for most of the individual phenolic acids and flavonoids identified in the courgette fruits within the study. This result suggests that the organic system as such does not guarantee the superior composition of crops, which depends, among other factors, on the type of fertilizer inputs used in the cultivation.

Even though plant genotype is well known to play an important role in determining the quality features of crops, the two courgette cultivars included in this study did not differ significantly in the contents of sugars and the tested bioactive compounds. However, the untargeted metabolomic analysis in this study allowed several metabolic features to be pointed out which significantly differentiated courgette fruits of different cultivars and grown in different horticultural systems. This suggests that metabolomics can be a potentially useful tool for the authentication of crops grown in organic production systems, as well as for the recognition of crops representing different genotypes.

Next to the qualities such as vitamins and other health-promoting compounds, sensory attributes belong to the key factors determining consumers’ preferences for foods. Some significant differences in sensory values were identified between the fruits representing the two cultivars or/and coming from the three horticultural systems compared in the present study. In particular, conventional courgettes were characterized by the most intensive peel color and aquosity, but at the same time were the least hard and firm compared to the fruits from the two organic systems. There was also a trend towards higher overall quality of the organically grown fruits.

To the best of our knowledge, this is one of the first reports on the chemical composition, untargeted metabolomics, and sensory characteristics of organic versus conventional courgette fruits.

## Figures and Tables

**Table 1:** Sensory attributes used in the quantitative descriptive analysis (QDA) of organic and conventional courgette fruits of Astra Polka and Nimba cultivar.

Odor Attributes		Taste Attributes		Appearance and Texture Attributes and Overall Quality	
Name	Abbreviation	Name	Abbreviation	Name	Abbreviation
Buttery odor	bo	Potato taste	ptt	Peel color	pc
Potato odor	pto	Sunflower taste	sft	Flesh color	fc
Sweet odor	so	Buttery taste	bt	Hardness	har
Sunflower odor	sfo	Sweet taste	st	Firmness	fir
Pungent odor	pno	Cucumber taste	ct	Aquosity	aqs
Earthy odor	ero	Bitter taste	btt	Fibrousness	fbs
		Pungent taste	pnt	Overall quality	ovq

**Table 2:** The main effect of, and interactions between, cultivar and horticultural system on the content of dry matter, and individual and sum of sugars (g/100 g f.w.) in courgette fruits.

Factor	Dry Matter	Sugars (Sum)	Fructose	Glucose	Sucrose
	Cultivar (CV)				
Astra Polka (AP)	4.42 ± 0.17 ^1^	3.88 ± 0.22	1.93 ± 0.12	0.89 ± 0.10	1.05 ± 0.10
Nimba (N)	4.16 ± 0.20	3.71 ± 0.26	1.76 ± 0.16	1.01 ± 0.11	0.93 ± 0.10
	Horticultural System (HS)				
Commercial organic fertilizer (BIO)	4.60 ± 0.26	4.24 ± 0.29 A ^2^	1.87 ± 0.15	1.36 ± 0.13 A	1.01 ± 0.12 AB
Manure (MAN)	4.39 ± 0.24	4.27 ± 0.31 A	2.12 ± 0.21	0.93 ± 0.12 AB	1.21 ± 0.12 A
Mineral (MIN)	3.96 ± 0.15	2.96 ± 0.15 B	1.56 ± 0.07	0.63 ± 0.06 B	0.77 ± 0.10 B
	ANOVA *p*-values				
CV	0.408	0.598	0.413	0.803	0.415
HS	0.355	0.038	0.146	0.093	0.118
CV × HS	0.942	0.825	0.997	0.539	0.945

^1^ Data are presented as means (average content from different fruit samples) ± SE; ^2^ Values in columns followed by different capital letters (A,B) are significantly different at the 5% level of probability (Tukey’s test).

**Table 3:** The main effects of, and interactions between, cultivar and horticultural system on the content of vitamin C (mg/100 g f.w.) and selected groups of phenolic compounds (µg/g f.w.) in courgette fruits.

Factor	Vitamin C (DHA + L-Asc)	DHA	L-Asc	Polyphenols (Sum)	Phenolic Acids (Sum)	Flavonoids (Sum)
	Cultivar (CV)					
Astra Polka (AP)	8.02 ± 0.72 ^1^	6.29 ± 0.72	1.52 ± 0.15	37.89 ± 3.02	33.47 ± 2.86	4.42 ± 0.42
Nimba (N)	7.60 ± 0.65	6.40 ± 0.60	1.21 ± 0.15	40.89 ± 3.53	36.64 ± 3.25	4.25 ± 0.47
	Horticultural System (HS)					
Commercial organic fertilizer (BIO)	7.61 ± 1.27	6.08 ± 1.28	1.12 ± 0.17	34.42 ± 2.81 B ^2^	31.19 ± 2.88 B	3.24 ± 0.41 B
Manure (MAN)	7.52 ± 0.84	6.27 ± 0.83	1.25 ± 0.16	53.83 ± 3.89 A	47.52 ± 3.81 A	6.31 ± 0.47 A
Mineral (MIN)	8.31 ± 0.46	6.60 ± 0.40	1.71 ± 0.19	30.79 ± 1.56 B	27.03 ± 1.36 B	3.76 ± 0.26 B
	ANOVA *p*-values					
CV	0.969	0.828	0.192	0.489	0.493	0.753
HS	0.872	0.925	0.128	0.086	0.119	0.025
CV × HS	0.818	0.868	0.449	0.780	0.792	0.655

^1^ Data are presented as means (average content from different fruit samples) ± SE; ^2^ Values in columns followed by different capital letters (A,B) are significantly different at the 5% level of probability (Tukey’s test).

**Table 4:** The main effect of, and interactions between, cultivar and horticultural system on the content of individual phenolic acids and flavonoids (µg/g f.w.) in courgette fruits.

Factor	Gallic Acid	Chlorogenic Acid	Caffeic Acid	*p*-Coumaric Acid	Ferulic Acid	Quercetin-3-*O*-rutinoside	Kaempferol-3-*O*-glucoside
	Cultivar (CV)						
Astra Polka (AP)	13.52 ± 1.17 ^1^	5.23 ± 0.59	2.99 ± 0.61	6.51 ± 0.61	3.51 ± 0.55	2.24 ± 0.26	2.18 ± 0.27
Nimba (N)	14.20 ± 1.37	5.79 ± 0.73	3.69 ± 0.79	6.49 ± 0.70	4.22 ± 0.55	2.09 ± 0.26	2.16 ± 0.27
	Horticultural System (HS)						
Commercial organic fertilizer (BIO)	13.09 ± 1.26 B ^2^	3.43 ± 0.48 B	2.42 ± 0.79	7.31 ± 0.94	2.43 ± 0.41 B	1.22 ± 0.18 B	2.01 ± 0.31 B
Manure (MAN)	18.59 ± 1.55 A	8.68 ± 0.73 A	4.81 ± 0.98	7.70 ± 0.76	6.42 ± 0.67 A	3.10 ± 0.31 A	3.22 ± 0.25 A
Mineral (MIN)	9.71 ± 0.81 B	4.10 ± 0.41 B	2.94 ± 0.63	4.63 ± 0.48	3.02 ± 0.30 B	2.43 ± 0.19 A	1.33 ± 0.13 B
	ANOVA *p*-values						
CV	0.656	0.275	0.537	0.863	0.283	0.624	0.942
HS	0.016	0.012	0.309	0.572	0.019	0.020	0.093
CV × HS	0.243	0.987	0.771	0.771	0.406	0.505	0.781

^1^ Data are presented as means (average content from different fruit samples) ± SE; ^2^ Values in columns followed by different capital letters (A,B) are significantly different at the 5% level of probability (Tukey’s test).

**Table 5:** The main effect of, and interaction between, cultivar and horticultural system on the content of carotenoids and chlorophylls (mg/100 g f.w.) in courgette fruits.

Factor	Carotenoids (Sum)	*β*-Carotene	Lutein	Zeaxanthin	Chlorophylls (Sum)	Chlorophyll *a*	Chlorophyll *b*
	Cultivar (CV)						
Astra Polka (AP)	1.21 ± 0.11 ^1^	1.10 ± 0.11	0.08 ± 0.00	0.03 ± 0.00	2.23 ± 0.18	1.71 ± 0.16	0.52 ± 0.03
Nimba (N)	1.19 ± 0.16	1.08 ± 0.16	0.08 ± 0.01	0.03 ± 0.00	2.01 ± 0.16	1.51 ± 0.14	0.50 ± 0.03
	Horticultural system (HS)						
Commercial organic fertilizer (BIO)	1.17 ± 0.14	1.06 ± 0.14	0.09 ± 0.01	0.03 ± 0.00	2.21 ± 0.22	1.70 ± 0.19	0.51 ± 0.04
Manure (MAN)	1.47 ± 0.20	1.36 ± 0.20	0.08 ± 0.01	0.03 ± 0.00	2.35 ± 0.24	1.80 ± 0.21	0.55 ± 0.03
Mineral (MIN)	0.96 ± 0.11	0.86 ± 0.11	0.08 ± 0.00	0.03 ± 0.00	1.84 ± 0.15	1.37 ± 0.13	0.47 ± 0.03
	ANOVA *p*-values						
CV	0.950	0.966	0.479	0.426	0.461	0.396	0.744
HS	0.557	0.568	0.514	0.443	0.409	0.313	0.642
CV × HS	0.978	0.977	0.999	0.973	0.866	0.748	0.942

^1^ Data are presented as means (average content from different fruit samples) ± SE.

**Table 6:** The main effect of, and interactions between, cultivar and horticultural system on the odor attributes of courgette fruits.

Factor	Buttery Odor	Potato Odor	Sweet Odor	Sunflower Odor	Pungent Odor	Earthy Odor
	Cultivar (CV)					
Astra Polka (AP)	1.70 ^1^ ± 0.29	2.96 ± 0.25	1.47 ± 0.25	2.04 ± 0.22	1.97 ± 0.23 A	1.74 ± 0.27 A
Nimba (N)	1.92 ± 0.29	2.57 ± 0.23	1.82 ± 0.27	2.39 ± 0.25	1.44 ± 0.20 B	1.05 ± 0.17 B
	Horticultural system (HS)					
Commercial organic fertilizer (BIO)	1.73 ± 0.31	2.28 ± 0.23 B ^2^	1.55 ± 0.26	2.36 ± 0.32	1.68 ± 0.26	1.37 ± 0.27
Manure (MAN)	1.78 ± 0.40	3.06 ± 0.28 A	1.69 ± 0.31	2.34 ± 0.28	1.67 ± 0.27	1.25 ± 0.22
Mineral (MIN)	1.93 ± 0.36	2.95 ± 0.33 AB	1.69 ± 0.38	1.94 ± 0.28	1.77 ± 0.28	1.57 ± 0.33
	ANOVA *p*-value					
CV	NS	NS	NS	NS	0.012	0.007
HS	NS	0.021	NS	NS	NS	NS
CV × HS	<0.001	<0.001	NS	NS	0.004	<0.001

^1^ The numerical values given are based on a 10-point scale and are presented as means (average evaluation score of different fruit samples) ± SE; ^2^ Values in columns followed by different capital letters (A,B) are significantly different at the 5% level of probability (Tukey’s test).

**Table 7:** The main effect of, and interactions between, cultivar and horticultural system on the taste attributes of courgette fruits.

Factor	Potato Taste	Sunflower Taste	Buttery Taste	Sweet Taste	Cucumber Taste	Bitter Taste	Pungent Taste
	Cultivar (CV)						
Astra Polka (AP)	2.97 ^1^ ± 0.22	2.57 ± 0.29	1.19 ± 0.25	1.63 ± 0.27 B ^2^	2.53 ± 0.21 B	1.08 ± 0.22	1.22 ± 0.26 A
Nimba (N)	2.66 ± 0.24	2.76 ± 0.28	1.26 ± 0.21	2.03 ± 0.30 A	3.05 ± 0.24 A	0.74 ± 0.15	0.76 ± 0.18 B
	Horticultural System (HS)						
Commercial organic fertilizer (BIO)	2.55 ± 0.26	2.98 ± 0.42	1.05 ± 0.26	1.81 ± 0.39	3.04 ± 0.33	1.01 ± 0.26	1.01 ± 0.28
Manure (MAN)	2.69 ± 0.31	2.68 ± 0.32	1.20 ± 0.26	1.73 ± 0.30	2.80 ± 0.24	0.71 ± 0.19	0.88 ± 0.25
Mineral (MIN)	3.21 ± 0.28	2.35 ± 0.31	1.43 ± 0.32	1.93 ± 0.38	2.53 ± 0.26	1.02 ± 0.24	1.08 ± 0.29
	ANOVA *p*-values						
CV	NS	NS	NS	0.046	0.037	NS	0.025
HS	NS	NS	NS	NS	NS	NS	NS
CV × HS	NS	NS	0.043	0.017	NS	NS	NS

^1^ The numerical values given are based on a 10-point scale and are presented as means (average evaluation score of different fruit samples) ± SE; ^2^ Values in columns followed by different capital letters (A,B) are significantly different at the 5% level of probability (Tukey’s test).

**Table 8:** The main effect of, and interaction between, cultivar and horticultural system on the appearance and physical attributes as well as overall quality assessment of courgette fruits.

Factor	Peel Color	Flesh Color	Hardness	Firmness	Aquosity	Fibrousness	Overall Quality
	Cultivar (CV)						
Astra Polka (AP)	7.10 ^1^ ± 0.26	4.78 ± 0.36	4.06 ± 0.33 B	4.36 ± 0.32 B	5.42 ± 0.30	2.68 ± 0.27	5.36 ± 0.22
Nimba (N)	6.45 ± 0.34	5.03 ± 0.41	5.12 ± 0.31 A	5.32 ± 0.32 A	4.95 ± 0.27	2.81 ± 0.30	5.57 ± 0.25
	Horticultural System (HS)						
Commercial organic fertilizer (BIO)	6.60 ± 0.34 AB ^2^	4.68 ± 0.50	4.96 ± 0.46 A	5.40 ± 0.41 A	4.83 ± 0.30 B	2.88 ± 0.37	5.57 ± 0.37
Manure (MAN)	6.34 ± 0.47 B	5.06 ± 0.49	5.06 ± 0.36 A	5.43 ± 0.35 A	4.67 ± 0.30 B	2.84 ± 0.32	5.69 ± 0.28
Mineral (MIN)	7.38 ± 0.28 A	4.98 ± 0.42	3.75 ± 0.35 B	3.70 ± 0.36 B	6.06 ± 0.39 A	2.51 ± 0.35	5.13 ± 0.28
	ANOVA *p*-value						
CV	NS	NS	0.010	0.020	NS	NS	NS
HS	0.035	NS	0.016	<0.001	<0.001	NS	NS
CV × HS	NS	NS	NS	NS	NS	NS	0.031

^1^ The numerical values given are based on a 10-point scale and are presented as means (average evaluation score of different fruit samples) ± SE; ^2^ Values in columns followed by different capital letters (A,B) are significantly different at the 5% level of probability (Tukey’s test).

## Data Availability

Data will be made available upon reasonable request by the corresponding author (Klaudia Kopczyńska).

## Supplementary Materials

The following are available online at https://www.mdpi.com/article/10.3390/foods10102475/s1.
