# Supplementary material for: Bioactive Compounds, Sugars, and Sensory Attributes of Organic and Conventionally Produced Courgette (Cucurbita pepo)
Source: Foods. 2021 Oct 15;10(10):2475. doi: 10.3390/foods10102475 (PMC8536166; doi:10.3390/foods10102475)
Supplement: Supplementary file 1 [file foods-10-02475-s001.zip › foods-1396857-supplementary.pdf]

foods MDPI

Supplementary Materials to the Article:

Bioactive Compounds, Sugars and Sensory Attributes of Organic and Conventionally Produced Courgette (*Cucurbita pepo*)

Klaudia Kopczyńska [1,*], Dominika Średnicka-Tober [1], Ewelina Hallmann [1], Jacek Wilczak [2], Grażyna Wasiak-Zys [1], Zdzisław Wyszyński [3], Katarzyna Kucińska [3], Aneta Perzanowska [3], Paweł Szacki [3], Marcin Barański [4], Paulina Gawron [1], Rita Góralska-Walczak [1], Ewa Rembiałkowska [1] and Renata Kazimierczak [1]

[1] Department of Functional and Organic Food, Institute of Human Nutrition Sciences, Warsaw University of Life Sciences, Nowoursynowska 159c, 02-776 Warsaw, Poland; dominika_srednicka_tober@sggw.edu.pl (D.Ś.-T.); ewelina_hallmann@sggw.edu.pl (E.H.); grazyna_wasiak_zys@sggw.edu.pl (G.W.-Z.); paulina_gawron@sggw.edu.pl (P.G.); rita_goralska_walczak@sggw.edu.pl (R.G.-W.); ewa_rembialkowska@sggw.edu.pl (E.R.); renata_kazimierczak@sggw.edu.pl (R.K.)

[2] Department of Physiological Sciences, Institute of Veterinary Medicine, Warsaw University of Life Sciences, Nowoursynowska 159, 02-776 Warsaw, Poland; jacek_wilczak@sggw.edu.pl

[3] Department of Agronomy, Institute of Agriculture, Warsaw University of Life Science, Nowoursynowska 159, 02-787 Warsaw, Poland; zdzislaw_wyszynski@sggw.edu.pl (Z.W.); katarzyna_kucinska@sggw.edu.pl (K.K.); aneta_perzanowska@sggw.edu.pl (A.P.); pawel_szacki@sggw.edu.pl (P.S.)

[4] Laboratory of Neurobiology, Nencki Institute of Experimental Biology, Polish Academy of Sciences, Pasteura 3, 02-093 Warsaw, Poland; m.baranski@nencki.edu.pl

* Correspondence: klaudia_kopczynska@sggw.edu.pl; Tel.: +48-225-937-037

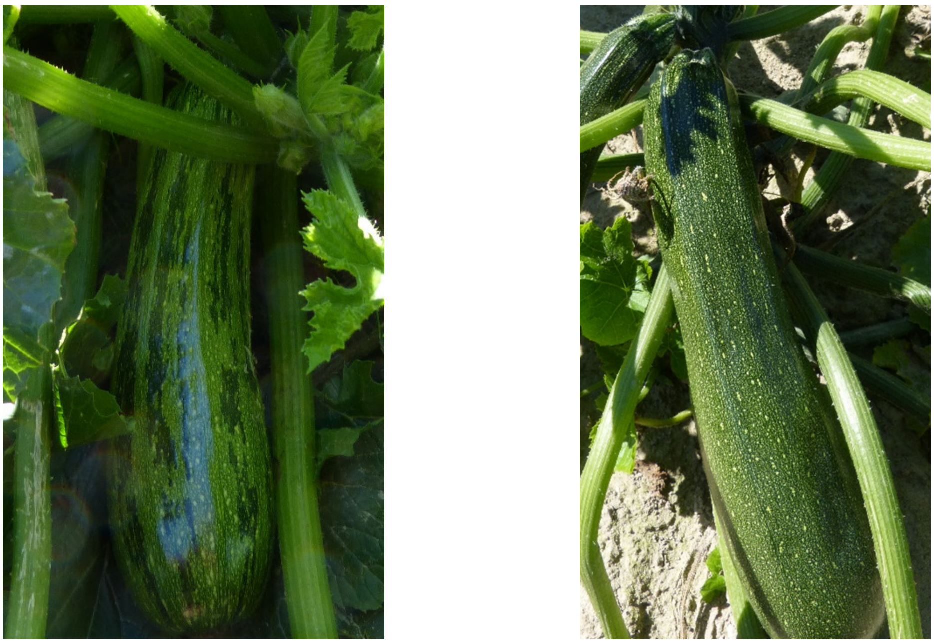

(**a**) (**b**)

Figure S1. Courgette fruits of two cultivar: (a) Nimba and (b) Astra Polka.

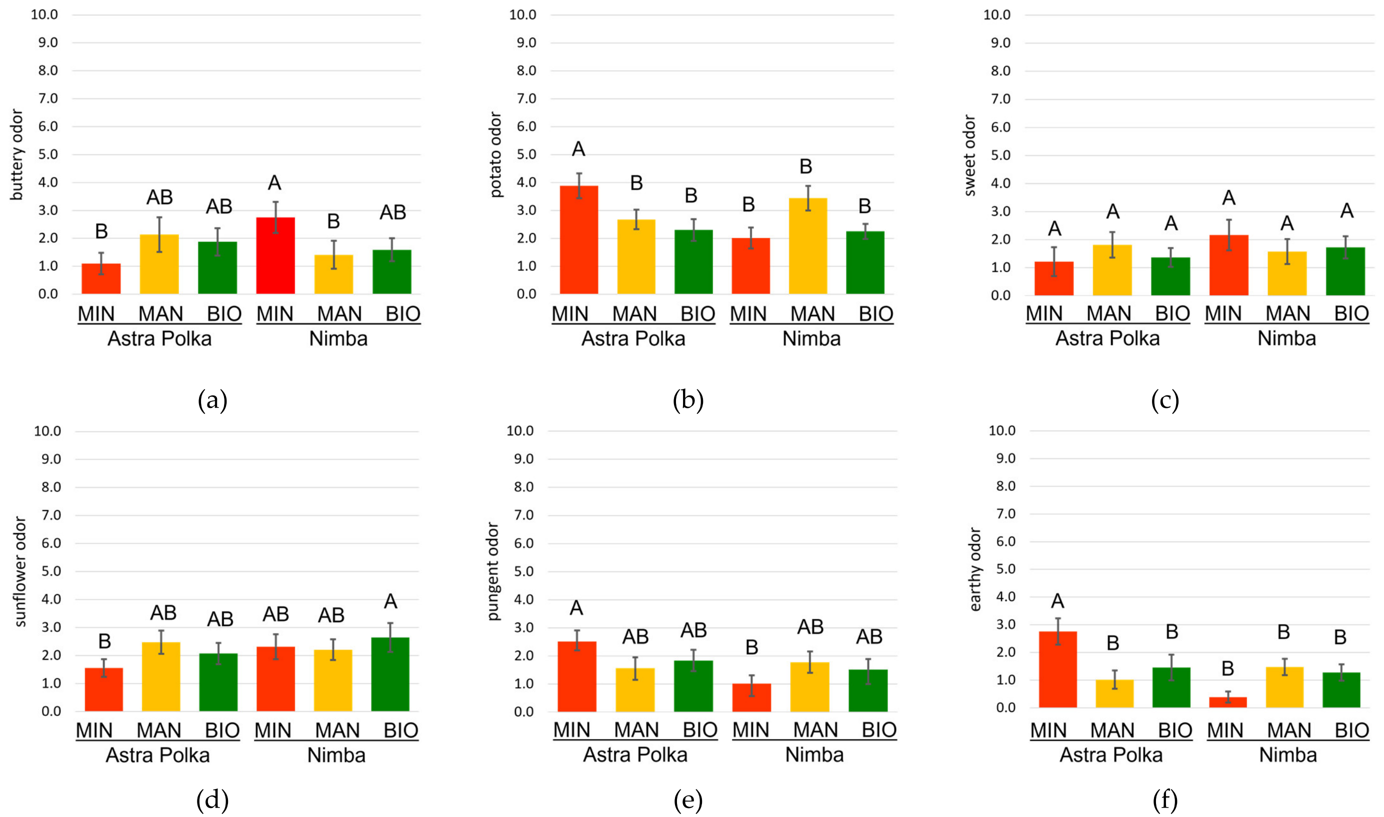

Figure S2. Odor attributes of the courgette fruits of Astra Polka and Nimba cultivars grown in the horticultural system based on commercial organic fertilizer (BIO), manure (MAN) or mineral fertilizer (MIN): (a) buttery odor, (b) potato odor, (c) sweet odor, (d) sunflower odor, (e) pungent odor and (f) earthy odor. Data are presented as means and standard errors. Within each figure, bars marked with the same capital letters are not significantly different at the 5% level of probability (Tukey's test between all cultivar-fertilizaton groups together).

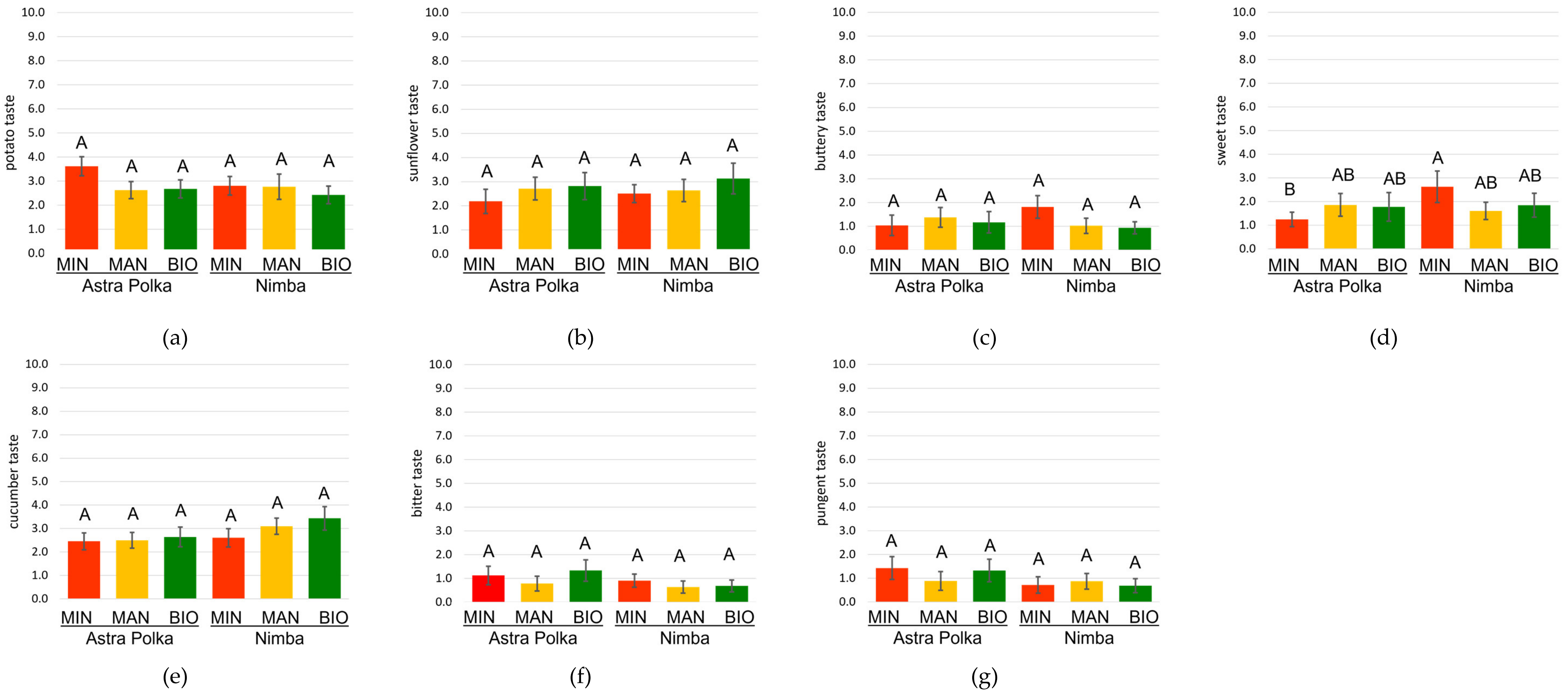

Figure S3. Taste attributes of the courgette fruits of Astra Polka and Nimba cultivars grown in the horticultural system based on commercial organic fertilizer (BIO), manure (MAN) or mineral fertilizer (MIN): (a) potato taste, (b) sunflower taste, (c) buttery taste, (d) sweet taste, (e) cucumber taste, (f) bitter taste and (g) pungnet taste. Data are presented as means and standard errors. Within each figure, bars marked with the same capital letters are not significantly different at the 5% level of probability (Tukey's test between all cultivar-fertilization groups together).

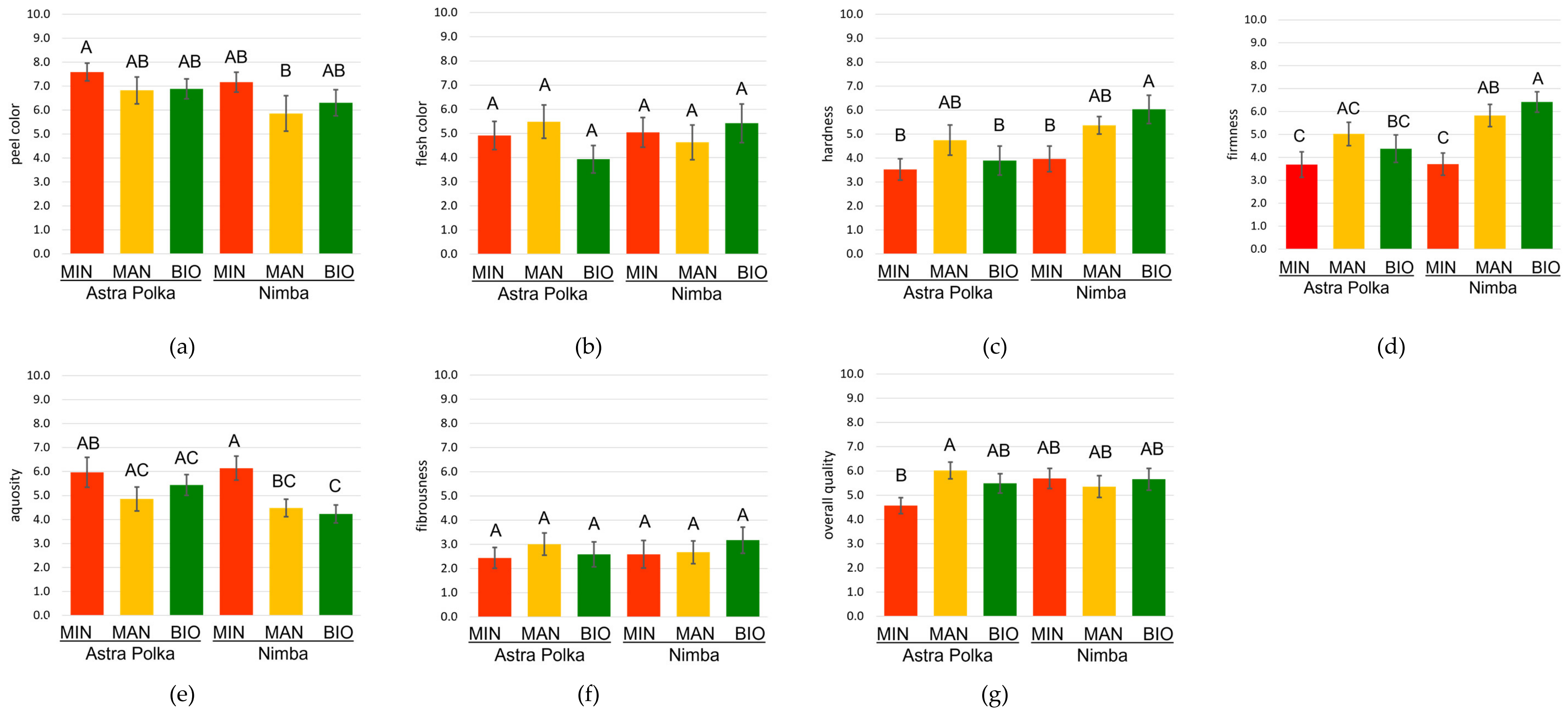

Figure S4. Texture and appearance attributes and overall quality assessment of the courgette fruits of Astra Polka and Nimba cultivars grown in the horticultural system based on commercial organic fertilizer (BIO), manure (MAN) or mineral fertilizer (MIN): (a) peel color, (b) flesh color, (c) hardness, (d) firmness, (e) aquosiy, (f) fibrousness and (g) overall quality. Data are presented as means and standard errors. Within each figure, bars marked with the same capital letters are not significantly different at the 5% level of probability (Tukey's test between all cultivar-fertilization group together).

Table S1. The retention time of the metabolic features, the ratio of the atomic mass to the number of elementary ion charges (m/z) and statistical parameters: t-value (coefficient indicating the direction of differences in the m/z values), p-value (the significance of differences in the m/z values), fold change (the ratio of the m/z values of a given metabolic feature) in fruits representing different cultivars and grown in different horticultural systems.

Retention time	m/z	t-value	p-value	Fold change
Compared samples: Nimba to Astra Polka				
1.67	188.0706	4.571	0.0006	3.53
36.59	542.3132	-3.762	0.0018	0.41
39.90	599.4278	-4.950	0.0005	0.31
40.85	306.2764	6.456	0.0000	4.52
40.85	284.2944	4.955	0.0003	2.64
43.43	567.4170	-4.215	0.0009	0.48
45.25	774.5569	-5.646	0.0002	0.17
45.25	653.4697	-3.776	0.0017	0.41
45.26	756.5466	-4.879	0.0009	0.12
48.13	754.5295	4.770	0.0003	2.31
Compared samples: conventional (mineral fertilizer) to organic (commercial organic fertilizer)				
29.07	359.2393	7.426	0.0004	5.46
38.62	353.2624	11.102	0.0000	8.70
39.32	573.4843	7.130	0.0004	7.12
43.71	693.4462	15.653	0.0000	16.32
44.90	393.2744	38.584	0.0000	243.30
51.39	693.4370	7.362	0.0001	3.60
51.83	668.6488	-5.532	0.0003	0.58
52.40	931.5735	-4.938	0.0007	0.54
53.33	575.4946	8.164	0.0000	5.45
53.56	791.6271	5.664	0.0006	5.36
Compared samples: conventional (mineral fertilizer) to organic (manure)				
23.43	349.1980	7.987	0.0002	23.62
29.07	359.2393	7.872	0.0004	6.51
36.97	353.2662	-9.542	0.0002	0.00
39.33	393.2399	-6.019	0.0002	0.18
41.82	551.4982	-10.052	0.0001	0.03
43.71	693.4462	15.833	0.0000	13.81
45.14	445.3641	-5.321	0.0004	0.56
46.37	714.5465	-5.389	0.0005	0.55
51.38	721.4680	-10.602	0.0001	0.05
51.39	693.4370	7.292	0.0002	3.28
Compared samples: organic (manure) to organic (commercial organic fertilizer)				
36.96	376.2514	5.333	0.0006	3.30
36.97	353.2662	6.243	0.0003	3.78
36.99	391.2155	6.233	0.0004	2.86
39.33	393.2399	5.239	0.0004	5.17
40.04	433.2291	5.420	0.0003	4.23
41.82	551.4982	8.002	0.0000	10.47
50.38	672.4640	4.668	0.0009	2.44
51.38	721.4680	4.835	0.0009	3.61
52.42	899.5592	-4.746	0.0013	0.35
53.79	837.6452	4.546	0.0011	2.01
